# “It Feels Like They Cut off My Wings”: Cervical Cancer Stories and Experiences Among Middle- to Older-Age Latina Survivors in the United States

**DOI:** 10.3390/ijerph23080992

**Published:** 2026-07-29

**Authors:** Cirila Estela Vasquez Guzman, Susan J. Rosenkranz, Carlos Martinez, Kyleigh A. Layman, Blair G. Darney

**Affiliations:** 1 Department of Family Medicine, Oregon Health & Science University, Portland, OR 97239, USA; 2 Division of Oncological Sciences, Knight Cancer Institute, Oregon Health & Science University, Portland, OR 97239, USA; 3 School of Nursing, Oregon Health & Science University, Portland, OR 97239, USA; rosenkra@ohsu.edu; 4 School of Medicine, Oregon Health & Science University, Portland, OR 97239, USA; marcarlo@ohsu.edu (C.M.); laymank@ohsu.edu (K.A.L.); 5 Guttmacher Institute, New York, NY 10038, USA; darneyb@ohsu.edu; 6 Department of Obstetrics and Gynecology, Oregon Health & Science University, Portland, OR 97239, USA; 7 Center for Population Health Research (CISP), National Institute of Public Health (INSP), Cuernavaca 62100, Mexico

**Keywords:** cervical cancer, Latinas, middle to older age, formative experiences, health equity, health disparities, health systems redesign

## Abstract

**Highlights:**

**Public health relevance—How does this work relate to a public health issue?**

- Latinas are more likely to receive a late cervical cancer diagnosis.
- Middle- to older-age Latinas are still at equal risk for cervical cancer, but we know very little about their experiences.

**Public health significance—Why is this work of significance to public health?**

- Most interventions target younger reproductive-aged individuals even though middle- to older-age individuals likely did not receive the HPV vaccination.
- We have a rapidly growing aging Latina population in the U.S. that can still benefit from timely cervical cancer screening and prevention.

**Public health implications—What are the key implications or messages for practitioners, policy makers and/or researchers in public health?**

- Healthcare systems and providers may need to better provide the language and support for middle- to older-age Latinas to not only advocate for themselves and reduce isolation but also to help inform the next generation of daughters, nieces, and aunts.
- More research is needed to prevent late diagnosis that contributes to and compounds a sense of urgency affecting both providers and patients’ communication and decision-making processes.

**Abstract:**

Background: Although cervical cancer is preventable, Latinas are twice as likely to receive a late diagnosis compared with non-Hispanic Whites (NHW). We currently know very little about the experiences of cervical cancer among middle- to older-age Latinas in the U.S. This study aimed to document Latina cervical cancer survivors’ journeys with an emphasis on improving the cervical cancer system of care to address inequities among a rapidly aging Latina population. Methods: We interviewed participants aged ≥ 40 years with a diagnosis of cervical cancer of three months or greater who identify as Latina. Recruitment was multi-pronged, including electronic healthcare records search, community tabling events, and snowball sampling. We conducted a qualitative study using the Database of Individual Patient Experiences (DIPEx) methodology and identified themes using grounded theory constructivist analysis. Results: A total of 20 Latinas (43 to 85 years old) participated, including 16 Spanish-language interviews. We identified three themes: formative experiences driving screening and prevention behaviors, provider communication that was suboptimal throughout the cancer continuum, and survivorship impacts including isolation and stigma with implications for the next generation. Conclusions: A central takeaway was the pervasiveness of Latinas’ feelings of shame, lack of communication, and isolation. Healthcare systems must improve culturally responsive communication, survivorship support, and screening equity for aging Latina populations. *Abuelas*, *tias*, *and*/*or primas* (grandmothers, aunts, and cousins) could play a critical role in raising awareness and increasing timely and early screening among the wider Latina population.

## 1. Introduction

In 2026, an estimated 13,490 people in the U.S. will be diagnosed with invasive cervical cancer, and 4200 will die from the disease [1]. Cervical cancer can affect a person with a cervix of any age and is very preventable with access to the human papillomavirus (HPV) vaccine and early/timely cervical cancer screening. However, Latinas experience a disproportionate burden of cervical cancer in the U.S. They have the highest incidence rates of any racial/ethnic group, nearly twice as likely to be diagnosed with cervical cancer compared with non-Hispanic whites (NHWs). They also have the second-highest mortality rates from cervical cancer following non-Hispanic Blacks. Latinas are 25% to 50% more at risk of dying than NHWs [2]. Thus, timely cervical cancer screenings are critical for Latinas, especially for middle- to older-age (over age 40) Latinas who may not have benefited from the HPV vaccine (first offered in 2006 in the U.S.), making regular cytology even more critical for detecting precancerous cells [3]. Early detection of cervical cancer significantly improves survival rates, with a 5-year survival rate at 91% when caught early, compared with 60% after the cancer has grown [4]. This is even more important now, given that COVID-19 disproportionately caused a sharper decline in screening rates among Latinas (39.27% drop in 2020) compared with NHWs (21.15%) [5] and rates have continued to lag with long-term consequences.

Latinos in the U.S. face barriers to receiving adequate and timely medical services, including cancer screening and preventive services, compared with NHWs. Lack of health insurance, fear of diagnosis, language differences, limited awareness, and systemic challenges such as fear of immigration, lack of transportation, and/or limited culturally sensitive providers all drive cancer-related inequities [6]. National guidelines have long recommended cervical cancer prevention and screening for individuals with a cervix starting at age 21, with screening occurring every 3–5 years depending on age and test type. Given persistent gaps in screening, the National Cancer Institute in 2019 created the ‘Last Mile’ initiative to ensure no one gets left behind, given that more than half of cervical cancer diagnoses occur in individuals who are infrequently or never screened for cervical cancer [7]. Additionally, the recent introduction of HPV self-collection in 2024 has the potential to address current barriers to screening but may also widen existing disparities among subgroups [7,8]. Although cervical cancer screening rates among Latinas are only slightly less than NHWs (71.6% vs. 74.6%, respectively) [9], there is heterogeneity within Latinas; cervical cancer screening rates among U.S. Latinas exhibit variability based on factors such as ethnicity, acculturation, and country of origin [10,11]. Foreign-born, uninsured, and/or recent immigrants (less than 10 years in the U.S.) are at highest risk for never having been screened, and immigrant Latinas are more likely than U.S.-born Latinas to have a late-stage cervical cancer diagnosis [12,13]. There has been a large body of literature focused on cervical cancer and younger reproductive age individuals, but there is a growing concern to focus on middle- to older-age individuals who remain at equal risk. We define middle age as spanning ages 40 to 64, while older age begins at 65 [14].

Very little research has focused on middle- to older-age individuals and cervical cancer, particularly among Latinas who are not only burdened unequally by cervical cancer but are also a rapidly aging population in the U.S. The U.S. Latino/a population reached 68 million in 2025, almost doubling in size since 2000. They make up 20% of the U.S. population, up from 14% in 2000 and just 6% in 1970 [15]. Latinos over the age of 50 are projected to grow from 4.6 million in 2019 to nearly 20 million by 2060 [15,16]. Cervical cancer frequently affects women in middle age, with a median diagnosis age of 50, and over 20% of cases occur in women over 65 [17]. Recent data suggests that middle- to older-age women are at heightened risk, often due to insufficient screening history in their earlier years, lack of access to HPV vaccination, and a weaker immune system [18]. Among the few studies conducted among this population, we know only 63.7% of older Latino/as (50–64) in a 2022 survey reported a recent Pap smear [19]. A growing body of international literature urges researchers to focus on middle- to older-age women to effectively address cervical cancer inequities, a group we argue remains at the margins.

The purpose of this study was to understand the cervical cancer experience—from screening to survivorship—among middle- to older-age Latinas to illuminate gaps and opportunities to improve the cervical cancer system of care for Latinas [7]. To fill this important gap in the literature, we leveraged an innovative qualitative approach to examine the complex, rich experiences of middle- to older-age Latinas diagnosed with cervical cancer.

## 2. Materials and Methods

We conducted a qualitative study utilizing the Database of Individual Patient Experiences (DIPEx) methodology—a time-tested, research-based approach for conducting and disseminating health experiences originally developed at Oxford University in 2001 [20,21,22]. The objective is to conduct and disseminate health experiences research using narrative as a resource in accounts of the experience of illness [23]. Fifteen additional countries have since implemented the DIPEx methodology, combining to form DIPEx International. This approach emphasizes the utilization of stories to improve care, thereby supporting experiences as evidence and informing decision-making [20]. All research activities were guided and informed by our 12-person community advisory board (CAB) that included Latina patients, as well as providers, community members, and medical students.

### 2.1. Teams Positionality and Background

Eight of the nine team members identified as female and seven identified as Latina/o. Our interdisciplinary study team included a medical sociologist and a medical anthropologist with over three decades of combined expertise conducting qualitative research, as well as two research assistants, two medical students, two undergraduate interns, and one high school student. Six team members were native Spanish speakers, two received formal training in Spanish, and only one was a monolingual English native speaker. Our bi-cultural, bilingual team enabled us to remain true to all participants’ original language and cultural background. The principal investigator who conducted all interviews is trained as a medical sociologist with expertise in qualitative methods, is a native Spanish speaker, was born in Mexico, and identifies as an immigrant Latina.

### 2.2. Eligibility

Participants were eligible to participate in the study if they were 40 years old or older, had been diagnosed with cervical cancer at least 3 months prior to enrollment, identified as Latina, and resided in the U.S. Criteria for exclusion consisted of anyone younger than 40, a cervical cancer diagnosis less than 3 months, identifying as non-Latina, and travels or lives a majority of the time outside of the U.S. No one was excluded if they had a chronic condition, cognitive limitations, or other concurrent cancers. This study specifically focused on the Pacific Northwest (PNW), including Oregon and Washington state. We applied strategies to achieve maximum variation in the sample that consider factors associated with key differences in patient experience (e.g., socio-economic status, language) to ensure that we capture a wide range of views (i.e., a “maximum variation”) [24].

### 2.3. Data Collection and Instrument Development

Our recruitment approach was multi-pronged. First, we used electronic health records at a major academic medical center to identify people who met our study inclusion criteria. Second, we developed culturally tailored Spanish and English language flyers to recruit at community events, disseminated them at 150 clinics in the PNW, and distributed them widely for patients to self-contact us for more information. Third, participants were actively recruited through healthcare providers who were referred to us via snowball sampling [25]. Fourth, a social media campaign was conducted via Instagram and Facebook during cervical cancer awareness month (January), with a focus on the PNW.

Per the DIPEx methodology, all participants’ interviews were video- and audio-recorded with their consent. Interviews were all conducted in the participant’s preferred language (English or Spanish) in three parts. The first part consisted of open-ended questions designed to give patients the opportunity to tell their story [26] without assuming or telling them where to start. They were instructed to take as much or as little time as they wanted. The second part consisted of structured follow-up questions about their entire cervical cancer journey: screening/prevention, diagnosis, treatment, impact, and survivorship. Finally, in the third part of the interview, participants were instructed to look directly into the camera and offer recommendations on how to improve early detection of cervical cancer and better support Latinas to four audiences: other Latinas, providers, family and friends, as well as partners. The interview guide was co-developed and pilot-tested with the CAB members to further refine the wording. The first few interviews led to ongoing edits of the interview guide to ensure we were being as culturally tailored as possible. Our process therefore enabled us to adequately capture the entire cancer continuum from diagnosis to survivorship.

### 2.4. Data Saturation and Reflexive Memoing

The study team carefully monitored data saturation through reflexive memoing to determine when to stop data collection efforts. During our regular weekly team meetings, we held discussions about what we heard to inform biography summary sheets for each of the participants. Per the DIPEx methodology, all participants have a biography that summarizes their data in one to three pages with a demographics summary, key highlights of their journey, and key quotes [22,27]. Simultaneously, we kept note of the background and representation of the participants enrolled to ensure maximum variation per the DIPEx methodology [23]. The team concluded data saturation was reached around 2/3 through the interviews when we started to hear similar themes and concepts emerge. The final three interviews served as confirmation, and when no new codes were identified, the data collection stopped. Thematic sufficiency was monitored by the PI and two other undergraduate assistants who were directly involved in the data collection efforts (scheduling, attending, taking notes, and debriefing after each interview). Many DIPEx pilot studies have approximately 12 participants. A sample of 20 is sufficient for data analysis given that national DIPEx studies often include 35 to 50 in total and ours focused on the PNW region [28,29].

### 2.5. Data Analysis

The average duration of interviews was 90 min. Audio files were transcribed verbatim in the language in which they were conducted. Each transcript was compared with the video recording by a bilingual member of the research team and verified for accuracy. Transcripts were then entered into qualitative data software, NVivo 14 (Lumivero, Denver, CO, USA), for coding and analysis. Our CAB helped co-develop and refine the coding book [30]. Six study team members were involved in the coding of all transcripts. Each transcript had a primary coder and a secondary coder. To further enhance confidence in linguistic rigor, Spanish language transcripts were coded by bilingual coders and all Spanish-to-English language translations were reviewed for accuracy by three bilingual study team members throughout the analysis process. All disagreements were discussed and resolved bilingually at the regular ongoing bi-weekly team meetings. Codebook and theme development decisions were documented in meeting minutes and reflexive memos maintained by the study team as an audit trail to help ensure data trustworthiness, credibility, and dependability. Attention was paid to emergent (i.e., unexpected) themes as well as those that were anticipated based on the literature review, using the method of constant comparison. A grounded theory constructivist approach [31,32] involved three coding stages: (1) Initial or open coding where we reviewed and coded responses in the composite document, (2) Intermediate or selective coding where we summarized our codes and collapsed them into categories, and (3) Advanced or theoretical coding to identify final themes. Multiple data sets produced throughout analysis—biographies, interview transcripts, codes, themes, study team and CAB meeting minutes, reflexive memos—were used to secure data triangulation.

## 3. Results

We interviewed a total of 20 middle- to older-age Latinas in the PNW. Our median age of diagnosis was 45, and about a quarter were diagnosed during late stages of cervical cancer. About two-thirds of the sample spoke Spanish, with one speaking an indigenous language; roughly two-thirds earned <$50,000 annually, and about one-third were unable to drive or lacked access to regular transportation. Although only a handful of the participants were undocumented at the time of study enrollment, almost 90% of the sample reported they were currently or formerly undocumented at some point in their lives (see Table 1). About 30% did not know their cancer stage, and among the half that did, 7 were in early stage 1, 6 were locally advanced, and one was advanced (see Table 2). Nearly two-thirds (70%) underwent partial or full hysterectomies, over half (60%, *n* = 12) received radiation, and finally, less than half (40%, *n* = 8) received chemotherapy (see Table 2).

We identified three overarching themes that help us understand cervical cancer experiences among middle- to older-age Latinas: formative experiences, provider communication, and survivorship impacts. Themes and sub-themes are presented below; additional exemplars are presented in Table 3. Participant quotes that have been translated from Spanish to English are indicated with an asterisk (*) after the participant ID number.

### 3.1. Theme 1: Formative Experiences

Formative experiences captured influences and experiences in early childhood and adolescence that affected adulthood prevention and screening behaviors. Middle- to older-age Latina participants often started the interview by sharing their childhood experiences to help set up their upbringing context that often illuminated less of a focus for cervical cancer screening in adulthood. Two sub-themes emerged: economic hardship and reproductive health communication.

#### 3.1.1. Sub-Theme 1: Economic Hardships

Most of the middle- to older-age participants identified economic hardship as a major challenge growing up that likely affected how and when they went to the doctor. Preventive care was seen as a luxury and was deprioritized over essential expenses like food or rent. For example, a participant shared:

This country has…in Mexico, unfortunately it’s not so much because people don’t want to go [but] it is because of the economic situation. In our country, unfortunately, the economy is in tatters. Yes, we barely have time, like, how shall I tell you? Grab, for a week’s meal [or] the doctor.

(ID 06)*

Due to financial hardships, many participants recalled that even when they became sick, they were forced to “tough it out” due to lack of healthcare affordability. They shared how it felt like a form of socialization during this generational upbringing:

Routine health maintenance wasn’t a priority in our family…getting sick, you just went in your bedroom [laughing] and toughed it out. Sounds so sad now. We didn’t go to the doctor’s unless we were bleeding, [laughing] needed stitches or were really dying.

(ID 19)

The economic reality that middle- to older-age participants endured during their formative years instilled a deep sense of inaccessibility to healthcare and routine screenings. Such experiences not only affected their early childhood and adolescent years but carried on into adulthood.

#### 3.1.2. Sub-Theme 2: Reproductive Health Communication

The second theme captures the lack of communication about reproductive health during formative years. For the purposes of this analysis, we used the definition from the World Health Organization (WHO): “a state of complete physical, mental, and social well-being and not merely the absence of disease or infirmity, in all matters relating to the reproductive system and to its functions and processes.” Cervical cancer is part of reproductive health as it pertains to reproductive organs [33]. Most middle- to older-age participants shared an absence of open communication about puberty, sexuality, and reproductive health with family. Regardless of generational differences, many described how their mothers did not talk about menstruation or sexual development, let alone any suggestion of gynecological visits. For example, we heard from middle- to older-age participants, “The moms of the past were very deprived of those things. I mean, no, they didn’t talk to you about this, about sexuality…about when it was going to take you: ‘Oh, look, when you’re so old your period is coming’. I remember my mom never spoke to me about it” (ID 04)*. Another shared a similar sentiment:

No, no, parents didn’t talk about any of that, like they were hiding it, they were ashamed, I don’t know. They never, never told me. When I got my first period, I was in middle school. And it wasn’t until a girl said to me, “Hey, you’re bleeding.” I got scared. I said, “Why?” She said, “It’s normal, isn’t it? Didn’t your mom tell you?” No, she didn’t tell me. And they got me pads and stuff, but my mom never talked to us about it.

(ID 01)*

The lack of communication shared by middle- to older-age participants was not just limited to menstruation but also to issues regarding sex and pap smears. As a result, many entered adolescence and adulthood without basic knowledge or awareness of their own bodies. Several participants described learning about reproductive health only after they were already sexually active and, in roughly half the cases, after they had already become pregnant. These narratives expose silence around menstruation and reproductive health, leaving many middle- to older-age participants without the language needed to self-advocate with providers in adulthood and the future.

Overall, participants described economic hardships and limited sexual health communication that did not encourage gynecological screening, did not inform language about their bodies, and shaped adulthood behaviors. Many reported not having a trusted source to ask questions and instead used the internet or just kept silent, emphasizing the need to change this for future Latinas. When asked what message they would like to share with other Latinas, all the participants urged Latinas to go to the doctor, to make time for it regardless of costs, to share everything, ask questions, and to prioritize all prevention and screening tools.

### 3.2. Theme 2: Provider Communication

Middle- to older-age participants also relayed that they experienced later diagnoses with less-than-ideal provider communication. Provider communication is the bidirectional exchange of information, emotions, and treatment plans between healthcare professionals and patients. The first sub-theme is Low Risk Perception, minimizing the need for cervical cancer screening and/or HPV testing. The second sub-theme, Delivery of Diagnosis, refers to the use of victim-blaming language such as “waiting” so long to get screened to receive a timely diagnosis.

#### 3.2.1. Sub-Theme 1: Low Risk Perception

Providers conveyed a message of little to no risk to middle- to older-age Latinas. Several participants reported that during clinical encounters they were advised not to pursue cervical cancer screening and/or HPV testing, with providers frequently citing patients perceived “low risk” as justification.

[My friend] said, “Has she ever checked you for, like, HPV or other STDs? And I said, “No. I’ve asked her before, and what she told me was, “you’re not high risk. You’re married. You only have one partner. You’ve been with this person for 16, 17 years. And you don’t have other partners.” I said, “No. Of course not.” And so she goes, “Yeah, you’re not really at high risk.” I went back because of my friend insisted that she check me for every STD possible, including HPV.

(ID 22)

Another participant also described perceptions of risk related to having a single sexual partner for nearly 20 years, which influenced how their provider understood their low risk for HPV. Providers did not recommend or offer screening for many of the middle- to older-age participants. Participants didn’t question or push back, “I didn’t question it. I didn’t think to” (ID 19).

#### 3.2.2. Sub-Theme 2: Delivery of Diagnosis

Middle to older Latina participants shared that providers used dehumanizing or blaming language during delivery of diagnosis. For example, a participant shared that during a hospital admission she was eating a burger in her bed when a doctor entered the room and said, “You know what? You have cancer” (ID 01)*. She shared how even her husband was taken aback by how abrasive the delivery of the news was and how the language felt blaming. Nearly all the Latina women shared how providers made them feel shamed for “waiting” this long. Not all deliveries of diagnosis were negative; in about half of the sample, providers were very supportive: “Oh, [the doctor] hugged me and said, ‘It’s all right—it’s going to be okay. Don’t worry. We are going to do what we have to do.’ She gave me a lot-a lot of encouragement” (ID 04)*.

Overall, Latinas received mixed or contradictory messages from providers—being told they were at “low-risk” while simultaneously being blamed for “waiting” to get screened. This suboptimal communication left critical assumptions unaddressed, particularly the recurring belief that a cervical cancer diagnosis was a death sentence. For example, one woman explained, “To me I thought I was going to die. Because from one, it’s like, it just happened boom, boom, boom, boom. And I thought to myself if he’s doing it that quickly, there must be some, you know, it must be bad” (ID 05). Another participant shared similar sentiments:

They spoke to me on the phone. Well, how do you feel? Well, you feel like you’re going to die. It is a word, like a sentence of death, when they tell you, “You have cancer.” “You have symptoms of cancer.” They tell you one, of those things and you say, “Well, it’s a death sentence.” Because you don’t know if you’re going to do it. And all your wings fall off and no matter how brave you are, there you say, well, you have to hold on to God’s hand, and He is the one who can hold you.

(ID 02)*

This concern could have been addressed by the provider or by creating space for the patient to ask questions, but instead many women shut down and carried this fear throughout their treatment and follow-up care. Many middle- to older-age participants had many remaining questions about their cervical cancer journey, what it was, how they got it, whether they could get it again, etc. In fact, they shared their story in hopes that the next Latina would have more information and understanding about their journey, leaving them to feel more empowered and less alone.

### 3.3. Theme 3: Survivorship Impacts

The third theme captured participants’ impact in the context of survivorship in middle to older age. Cancer survivorship impact refers to the comprehensive, long-term physical, psychological, social, and economic consequences experienced by a person from the moment of a cancer diagnosis, through treatment, and for the rest of their life. We identified three sub-themes: first, Isolation: during and even after surviving cervical cancer, there was a need for stronger support systems; second, Stigma of the cervical cancer diagnosis that led to further silence; and third, Communication between generations: the missed opportunity to inform and share with the next generation.

#### 3.3.1. Sub-Theme 1: Isolation

During diagnosis and treatment, 16 women shared their diagnosis with their partner, friends, or family. However, most shared it with only a few close people (e.g., partner, immediate family member, or a close friend) and kept their diagnosis a secret from other family members and friends. For some who elected to share their diagnosis, relationships changed, and people became distant: “I felt that [my cancer] was something like with certain people, like contagious […] And there were people who I felt were moving away from me” (ID 20)*. Another woman described a lack of support from close friends as she went through treatment: “Um, nobody drove me. Nobody was going with me holding [my hand]. None” (ID 14). In two cases, women kept their diagnosis to themselves, despite their cancer being diagnosed nearly a decade ago. Although they eventually revealed their cancer diagnosis to select people, they did not reveal that it was cervical. One shared, “No, because I don’t trust much in, in the, in telling my things, or to friends, or to family either. I’m very reserved. My sisters tell me, they are very gossipy. They tell each other everything” (ID 01)*. The other woman said, “Even to my mom, I didn’t tell her what—everything I was going through. I locked myself in. I didn’t want anyone to see me. I went through that process. I didn’t want anyone to see me” (ID 06)*. Keeping their diagnosis secret was isolating and limited access to systems of support from family, friends, and community. Upon reflection, several women felt they may have benefitted from having support at the time of receiving a diagnosis, as one woman explained:

One doesn’t realize how much support they need at that time and how out of body they could get. I certainly didn’t. And I needed that support more than I understood what support even meant, more than I understood what support meant in my entire life.

(ID 14)

#### 3.3.2. Sub-Theme 2: Stigma

Second, several participants shared a persisting sense of shame. The formative experiences of middle- to older-age Latinas may have contributed to the taboo of discussing cervical cancer diagnosis. HPV is a sexually transmitted disease, and many participants felt shame when sharing their history with sexual partners. “When that happened to me, I felt very ugly…well, I’m not married, right? We’re just together or I don’t know how to tell him” (ID 17)*. Another Latina shared that HPV was often associated with women who had multiple sexual partners:

That’s some type of stigma. I kinda felt that way…it is called the dirty woman’s disease. And I [laughing] that’s not true, no. But this stigma of it could back a long time ago. That’s what it was associated with. A woman who was dirty. And they meant dirty by she had sex with a whole bunch of different men.

(ID 22)

In many cases, that stigma of HPV carried over into their personal lives and relationships. For one participant, this further influenced their actions and she ceased any sexual interactions or relationships, “And I don’t want to have sex anymore, no, no. Because it was based on that…That’s how I got infected” (ID 20)*. Another shared:

I was abstinent afterwards. I wasn’t with anyone…that was a big impact…just knowing the history of HPV in the past. What it was called and who was related to- it was like women from the streets that had that kind of disease. And, I’m not a woman of the street…usually it was associated with prostitution and so forth. That’s who had that disease.

(ID 22)

In some cases, women shared their history, but the majority decided to no longer engage in any kind of sex, ever.

#### 3.3.3. Sub-Theme 3: Communication Between Generations

We found that most Latinas lacked knowledge about their own family cancer history and were hesitant to share their diagnosis with family. In fact, cancer in general was not talked about amongst family members. We heard, “But it’s hardly ever talked about until it happens to you… Many people have cancer, but until it affects you or your family, you just hear, ‘Oh, so-and-so has cancer’” (ID 01)*. The lack of conversation surrounding cancer, especially cervical cancer, was shared by many participants. One Latina shared her hesitancy with sharing with her young children but received encouragement from her social worker husband, who noted the importance of preparing them:

I wasn’t going to say anything. Because I wanted to avoid causing suffering to my children… The first person I told and shared this with was my partner… It’s like when I received the news about my mother. It was so traumatic. It was so painful; it was terrible for me. Then, my partner, since he’s American [and] a social worker… he made me see that I have to think about them.

(ID 13)*

Ultimately, her decision to share her diagnosis with her children promoted more open and informed communication within her family. While many participants expressed that they did not want to share with their children to protect them, it often led to isolation and prevented further opportunities for education and conversations about cervical cancer. Another participant shared how little she knew about cervical cancer: “So I do have one question. Is it hereditary?” (ID 04)*. Despite this individual and two of her family members having cervical cancer, she still did not fully understand what caused cervical cancer. This sentiment was shared by other participants; the basics of cervical cancer are still not well understood among Latinas with personal experience and being survivors of cervical cancer.

## 4. Discussion

Cervical cancer is preventable, but there remain significant disparities, particularly among Latinas in the U.S with variation by language, foreign-born status, among other characteristics [1,2,3,34]. Very little cervical cancer research, however, has focused on people with cervixes outside of their reproductive years who may be at higher risk because they did not have the opportunity to receive the HPV vaccination, may have a less than complete screening history, and have declining immunity [18,19] In this paper, we investigated the lived experiences of middle- to older-age Latinas in the PNW to better understand their journey to address cervical cancer inequities. We found formative experiences driving screening and prevention behaviors, provider communication that was suboptimal throughout the cancer continuum, and survivorship impacts including isolation and stigma with implications for the next generation. These three themes are all key areas of needed additional attention and support by the healthcare system to improve cervical cancer inequities among middle- to older-age Latinas.

First, there is already a large body of literature documenting the importance of early life experiences and the impact they have on adulthood and later in life [35]. In fact, we found that middle- to older-age generations often experienced upbringings where sexual and reproductive health was considered taboo. This is not new in the literature [36]; however, we found middle- to older-age Latinas perceive that such socialization and lack of exposure affected their engagement with cancer screening prevention tools later in life. It was interesting to note that when invited to share their stories freely, many of them revealed how they grew up knowing very little about cancer prevention, reproductive health, and risk levels. These early experiences affected their adult cancer screening and prevention behaviors in a negative manner. Although well documented in the literature [34], this pattern remains understudied among an older age Latina population in the context of cancer screening. There is a substantial body of literature highlighting the relationship between adverse childhood experiences (ACEs) and early life circumstances on care across a range of diseases and conditions [35,37]. ACEs are linked not only to negative health outcomes but also to patterns of healthcare access and utilization [38]. Efforts by the CDC, NIH, and even the Affordable Care Act have advanced understanding of ACEs to support more tailored care; however, most research has focused on adolescents and early adulthood [39,40].

Similar efforts need to be made for middle- to older-age adults who have unique social-cultural-contextual upbringings that may inform cancer screening decision-making. Current sociocultural factors affecting colorectal, breast, and cervical screening are well-documented [41], but much less is known about earlier formative experiences. Considering middle- to older-aged Latinas’ childhood and formative experiences may help primary care providers and the broader cancer care system develop more responsive approaches to supporting cancer screening… Regardless of the large age differences, those raised in the 50’s vs. those raised in the 70’s or 80’s, communication on sexual and reproductive health was similar, suggesting little effect of generation context during formative experiences [42]. We urge researchers to focus on and systematically document middle- to older-age Latinas’ formative experiences around reproductive health to better inform healthcare practices and to increase awareness among healthcare providers.

Second, we currently know a lot more about provider communication during and post-cancer treatment [43], but less is known about provider communication during diagnosis aside from urging early and timely diagnosis [44,45]. In this study, diagnosis delivery, particularly in the emergency room, often occurred in suboptimal ways, negatively impacting Latinas’ cervical cancer journey. However, such suboptimal provider communication may not reflect the training or emotional capacity of the provider but rather the context in which screening is occurring. Significant research already exists on late diagnosis of cervical cancer often occurring in emergency rooms, mainly from large-scale survey data [46,47]. While 91% of respondents perceived delivering bad news as a very important skill, only 40% felt they had the training to effectively deliver such news, especially in challenging contexts [48]. Providers who are delivering the news in the emergency room are often overwhelmed and are frequently not the primary care provider of the patient [49]. Less has been studied in relation to cancer diagnosis; thus, we urge more attention be paid to such critical points in most patients’ cancer journey experience.

Notably, medical education training is not well versed in having complex conversations or delivering difficult news such as cancer diagnoses to patients, particularly for conditions where there are prevention and screening options [50]. It is important to continue to work towards a system that minimizes the conditions that lead to cancer diagnosis delivery in emergency rooms, a reality for many racial/ethnic populations, those without access to a primary care provider, and/or those with social–economic challenges [51,52].

Furthermore, in line with previous research, participants described experiences of isolation, suggesting that social support may be an important component of care for middle- to older-aged Latinas with cervical cancer. Social support has been a key concept in the larger research literature for decades [53], and is critical for cancer patients, with new evidence showing a direct impact on mortality rates [54]. As initially proposed by Berkman and Syme in 1979, social isolation is defined as a state characterized by the absence of social networks and support [55]. Additionally, recognition of the role of the health care system in addressing social isolation and loneliness is not new. In 1985, Jones et al. drew attention to the role of physicians in addressing loneliness: “General practitioners have unique opportunities to reduce the suffering caused by loneliness [56]”. We found a very high degree of social isolation among middle- to older-age Latinas with cervical cancer, largely related to multiple factors including silence around their own bodies, shutting down communication with providers, and not sharing their diagnosis with family or children who were often their main sources of social support. There is some literature connecting isolation and sharing cancer diagnosis [57,58], but less attention has been paid to cervical cancer. Among breast cancer patients/survivors, there is a greater tendency to share more openly with children and others, likely due to the lack of associated stigma [59].

These findings suggest that healthcare practitioners and the broader healthcare system could consider providing culturally and linguistically appropriate resources to help Latinas describe changes in their bodies and communicate about their experiences with others. Unlike breast cancer, cervical cancer is associated with sexual behavior—increasing shame or stigma despite the fact that more than 90 percent of sexually active men and 80 percent of sexually active women will be infected with HPV in their lifetime [60]. Again, it may be the context of a late diagnosis that contributes to the de-prioritization of education, but we argue in these cases that patient education may be even more imperative. In two cases, the interviewer was the first person the participant shared their cervical cancer experience with, even after being diagnosed nearly a decade ago. For many women, sharing this experience felt recent; they still had numerous questions regarding cervical cancer broadly and specifically, what was transpiring within their bodies. The intensity of the experience—even a decade later—underscores the need for the healthcare system to reflect on its role in silencing cervical cancer patients.

This study has limitations worth noting. First, this study’s generalizability is limited. The group we focused on was middle- to older-age Latinas, and our sample was limited to the PNW population. This focus allows our findings to have rigor with meaningful implications for a certain group, but it may not necessarily reflect the experiences of all Latinas across the U.S. A larger body of literature has focused on the East Coast, and future studies may benefit from expanding geographical representation of the study’s sample. These themes were not necessarily specific to the PNW and could have implications for the broader middle- to older-age Latina population in the U.S, although notably there are more Mexicans in the PNW compared with Dominicans or Puerto Ricans on the East Coast. Second, there may be some recall bias. Nearly half of the participants received their cervical cancer diagnosis more than a decade ago. However, the vividness and details they shared demonstrate just how raw such experiences remained for them. Nearly all of them demonstrated some level of psychological distress during the interview, and the interviewer took appropriate measures to ensure pausing and time to regroup. Retrospective self-report measures are subject to well-documented memory distortions that may compromise the accuracy of recalled cancer experiences. Reconstructive memory processes mean that recollections are not simply retrieved but actively reassembled at the point of recall, shaped by current emotional states, subsequent life events, and prevailing narrative schemas about illness [61,62]. This is particularly salient in cancer survivorship research, where the emotional intensity of diagnosis, treatment, and prognosis discussions confers heightened salience on certain memories, often rendering them more vivid, durable, and resistant to decay than mundane or procedural details [63]. This emotional response was much stronger in most interviews, with less ability to recall specific parts of their cancer journey. Long-term recall may disproportionately amplify emotionally charged or traumatic aspects of the cancer trajectory—such as the moment of diagnosis or acute symptom crises—while peripheral or affectively neutral information, including routine appointments, medication regimens, or day-to-day functional experiences, becomes increasingly attenuated or lost over time [63]. This selective amplification reflects the adaptive function of emotional memory in prioritizing threat-relevant information, but it simultaneously introduces a systematic bias whereby retrospective accounts may overrepresent distress and underrepresent the full complexity of the illness experience. Our research was concerned with improving future Latinas’ cancer experience across the continuum and less concerned with the specific details of their journey. Our findings still provide meaningful and useful insight into the experience and remain critical information to improving the next generation of Latina cancer patients.

## 5. Conclusions

This study addresses the National Cancer Institute Last Mile Initiative to ensure that all people are included in cervical cancer prevention, emphasizing middle- to older-age Latinas. Furthermore, research focuses on one aspect of a cancer story, but there is a larger literature emphasizing the need to understand the entire cancer continuum before diagnosis and even after treatment. This study focused on middle- to older-age Latinas’ journeys from prevention through survivorship. A central takeaway was the pervasiveness of participants’ feelings of shame, lack of communication, and isolation through formative experiences, provider communication, and extending into survivorship. While it is important to understand the immediate clinical scientific aspects of cancer treatment, it is also critical to address the long-term tangible complex experiences of cancer patients’ lived experiences of survivorship. The healthcare system can further help to mitigate isolation and increase capacity for sharing as well as support a healthy sexual life. When middle-aged and older Latinas do not disclose their diagnosis or talk about their experiences, valuable firsthand knowledge is lost. Younger generations miss the opportunity to learn from those who have already navigated similar challenges. Abuelas, tias, and/or primas (grandmothers, aunts, and cousins) could play a critical role in raising awareness and increasing timely and early cervical cancer screening among the wider Latina population.

## Figures and Tables

**Table 1:** Demographic characteristics of participants.

Characteristic	Participant ^a^ *n* (%)
Average age at time of interview, median	58
Range	(43–85)
Average age at time of diagnosis, median	45
Range	(27–65)
Date of diagnosis	
1990–2010	8
2011–2022	12
Ethnicity	-
Latinas	20(100)
Birth country	
Mexico	14 (70)
Central and South America	3 (15)
United States ^b^	3 (15)
Citizenship	
U.S. citizen	6 (30)
Legal Permanent Residents	6 (30)
Undocumented	4 (20)
Unknown/Not reported	4 (20)
Language of interview	
Spanish	15 (75)
English	5 (25)
Marital status at time of diagnosis	
Single	3 (15)
Married	8 (40)
Divorced	4 (20)
Partnered	2 (10)
Widow	3 (15)
Has children	20 (100)

^a^* N* = 20 participants, ^b^ United States included: California (1), New Mexico (1), Texas (1).

**Table 2:** Cervical cancer stage and treatment.

		Treatment				
Cervical Cancer Stage at Diagnosis	Participant ^a^ *n* (%)	Hysterectomy Only *n*	Radiation Only *n*	Radiation and Chemotherapy *n*	Hysterectomy and Radiation *n*	Hysterectomy, Radiation and Chemotherapy *n*
Stage I	7 (35)	3 (2 = partial; 1 = full)	1	1	0	2 (hysterectomy type unknown)
Stage II	2 (10)	0	0	1	0	1
Stage III	4 (20)	2 (1 = partial; 1 = full)	0	0	1 (full)	1
Stage IVB	1 (5)	0	0	1 (including brachytherapy)	0	0
Unknown/not reported	6 (30)	3 (2 = full; 1 = type unknown)	1	1	1	0

^a^* N* = 20.

**Table 3:** Themes, Definitions, and Examples.

Theme	Definition/Exemplar
**Formative** **experiences**	**Formative experiences captured early childhood and adolescence influences and experiences that affected adulthood prevention and screening behaviors.**
Economic Hardships	“We never, ever went to the doctor. And really too, about the bills, always. Because you say, ‘I pay the bills, or we eat.’ That’s what one bases on.” (ID 16) * Interviewer: “And how often did you go to the doctor in Mexico, or was it the first time you had a checkup?” ID 11: “Ah, almost not- I was…it was poverty with my family […] I come from a very, very humble family. Very poor. Sometimes we had nothing to eat. Ah, my dad, sometimes I didn’t have a job. My mom too. We were a family—a family of 12 siblings and very poor.” (ID 11) * “No, when I was a child, I only went to the doctor if I got sick.” (ID 22) “[My husband] stopped working for three months; Unfortunately, we couldn’t find help. I was going to lose my house, because we didn’t know how to get the aid that was supposed to be there, but no one offered it to us. So, I didn’t suppose—my husband—as I was in shock, we couldn’t do anything.” (ID 08) * “Well, with the expenses, really, what the insurance covered, and the other, they kept sending me as the payments. They have continued to send me the payments, but as I told them, I am going to pay as little as I can. Because I don’t have the money to pay for it either, but as long as we can, we’re going to pay.” (ID 17) *
Reproductive health communication	“No, no, well, since, as you say, before the parents didn’t talk about it at all, like what did they hide that, they felt sorry for it, I don’t know. I was never, never told. When I got my first period, I was in high school. And until a girl said to me, “Hey, you have blood.” I was scared. I said, “What about that?” She says, “That’s normal, didn’t your mom tell you?” No, she didn’t tell me. And they already got me towels and stuff, but my mom never talked to us.” (ID 01) * I met my first husband when we were 14 years old, and we were high school sweethearts. We were our one-and-onlies. We had a baby when I was 16 and we moved out towards the end of high school and I graduated high school. And we had our second baby when I was 19 years old. I would say through the process of being a young teen mom at that time, there was definitely a lack of support and understanding what was going on with my body, understanding pregnancy, having access to help take care of the babies. I figured it out. [Laughing] But, yes, even just as a young mom, having-having babies, it was difficult having support… I was so busy taking care of the kids, I really wasn’t paying attention to my body. I only went to the doctor when I was sick or- I always took my kids to the doctor. [Laughing] So I was just a busy, busy mom and I didn’t have any idea that screenings were so important. (ID 19) “And I just- I’m so overwhelmed with- I don’t know what to call my body parts, and how wrong it felt. Just mind blowing to me. I don’t have any idea where to put this information I have.” (ID 14)
**Provider Communication**	**Provider communication is the bidirectional exchange of information, emotions, and treatment plans between healthcare professionals and patients.**
Low Risk Perception	“So there was nine years that I didn’t go see the doctor ‘cause you have to- I always thought you had to be sexually active to have to go see ‘em, and when you’re not, then why go?” (ID 22) “They said, ‘Do you have any other partners or, you know?’ And I said, ‘No, it’s just me and my husband all our lives, you know.’ And she said, ‘Okay, well you don’t have to get a screening for another five years.’ So I just- I didn’t question it. I didn’t- yes. I didn’t question it. I didn’t think to because I thought it was every year, and I’ve already felt like I was behind. [Laughing] So I just didn’t question it.” (ID 19) “First, I was going with my appointments and I had changes in my body. In my body, I started to see heavy bleeding. Why? I went to my doctor. [They said,] ‘Okay, you’re already 40, you’re going to go through menopause.’ I started to feel symptoms in my belly. And I had never had strong back pain in my period, like when it opens up, that you are going to have a baby. And then I said, ‘That’s weird.’ So, I went back to my doctor. And my doctor told me not to worry, that it was normal, that it was because of pre-menopause.” (ID 08) * “So it was so bad. It hurt so bad. When I walked out, I had so many cramps. I was bleeding profusely. It was awful. So I waited. I think, about couple days later I got a phone call, and they told me that it didn’t seem that it was that bad. So we’ll just follow up with the next Pap in three months. Meanwhile, that was- um, so maybe- I don’t know how long after that I was having excruciating pain. So I called them, and I told ‘em. I let them know what was happening. And they said that they don’t think it’s cancer because cancer doesn’t hurt. For me not to- whatever. I knew there was something wrong.” (ID 05)
Delivery of Diagnosis	“’Ma’am,’ they told me, ‘you do have the neck cancer of the womb neck.’ As I tell you that I went on the wall [and] I fell. Then, my husband grabbed me. I felt like they threw a bucket of cold water on my head […] because I’ve heard […] there are times when there’s no cure, well. That’s what I thought. ‘I’m going to die, then.’ No-no-I couldn’t even find what to do with it. No. I was like, traumatized…” (ID 17) * “I went to one doctor I can’t remember why I switched. Of course, I didn’t go back to him afterwards, but he made me feel like I was like filthy dirty, just- I don’t know why I’d keep having these problems, whatever. He just literally made me feel awful. I’m like, ‘I’m never coming back to you.’” (ID 14)
**Survivorship Impacts**	**Cancer survivorship impact refers to the comprehensive, long-term physical, psychological, social, and economic consequences experienced by a person from the moment of a cancer diagnosis, through treatment, and for the rest of their life**
Isolation	“I came anyway, I didn’t speak, I didn’t say anything. I didn’t want to worry them more than they already were and when I came back I told my husband because he was… He stayed here and told me that it wasn’t right, that I was late to tell him, and I told him, but I didn’t have the courage to tell them, I didn’t tell them anything. And he told me well it’s okay since you didn’t say anything because it’s okay but I came anyway right, this is such an ugly pain hey that I don’t wish anyone on it, very ugly at the same time that nothing like something blames you blames you like it eats you, little by little it eats everything inside, It’s very ugly.” (ID 11) “When, when I went through the whole [treatment] process and everything, because no one knew. No, no one knew, only my sister and my neighbor knew, but many people didn’t know, and now that I’m cured and all that, I talk about it…” (ID 12) * “Uh, I didn’t want to tell my family; I didn’t want to talk to them because I said, well, it’s better that I stay there and not have like my family to be sad about what maybe they’re going to go through or I’m going to go through. But when I was alone, I would get sad and cry when I was bathing because I thought that that was it, that was the end of my day. When my doctor spoke to me, she was talking to me and said, ‘You have to make a decision right now because if it’s too late, we’re not going to be able to do anything for you.’” (ID 16) * “[A]t that time, too, I had not had supportive- knowing any other woman that had cervical cancer, at that point. So, it was extra scary.” (ID 14)
Stigma	“No. They soon realized for the same reason that my family said that I was bad, that I was very bad with cancer and then they saw that I was going to radiation all week and well, that’s it, but many people told me that- well, they did the fuchi, they were disgusted by me.” (ID 03)* “Where did this come from? Why did I get this? And-and just knowing the history of HPV in the past. What it was called and who was related to- it was like women from the streets that had that kind of disease. And I’m like, [laughing] I’m not a woman of the street. I’m not a, like a- usually it was associated with prostitution and so forth. That’s who had that disease. And so that made me feel, like, that is just not fair [laughing].” (ID 22)
Communication between generations	“No. And ‘what do you have mommy?’ and ‘what do you have?’, ‘I’m sick son’, women’s diseases, I never said anything to [my kids] even when they were older since everything was taken away from me. …They didn’t even know what, I was leaving my house because all from Monday to Friday, one of my daughters was fifteen years old, one of the youngest and she went to school, made them food, washed their clothes but not even she knew I was this sick, they knew I was sick but they didn’t know [from] what.” (ID 03) * “Well, I still kinda wonder because they always told me this- cancer doesn’t hurt. So to me I still wonder. I still wonder was it cancer or just having cramps to have cramps? I don’t know.” (ID 05) “But I certainly became a different kind of parent with [my daughter] and her girlfriends and giving them all the, you’re making choices by not making choices talks, and you should be talking to your parents or not at planned parenthood, and yada da da da, kind of conversations.” (ID 14) “I [have] something similar or the same with my mom who I never talk to [my daughters] about sex. I never talk to them about things like, ‘Look at this; of your sexuality.’ Not things like that.” (ID 16) * “And I remind my daughters all the time, [laughing] ‘Get your Pap.’ [Laughing] ‘Have you gotten your Pap this year?’ I’d never want them to feel the way I did.” (ID 19)

* Indicates participant quotes that have been translated from Spanish to English.

## Data Availability

Our data were collected for the research study and are not publicly available to protect study participant confidentiality and due to IRB and HIPAA restrictions. Requests to access the datasets should be directed to the corresponding author.
